# An Evaluation by Elderly People Living at Home of the Prepared Meals Distributed by Their Municipality – A Study With Focus on the Swedish Context

**DOI:** 10.5539/gjhs.v7n3p59

**Published:** 2014-10-28

**Authors:** Oleg Pajalic, Zada Pajalic

**Affiliations:** 1 Chalmers University, Gothenburg, Sweden; 2 Faculty of Health Sciences, Department of Health, Nutrition and Management, Oslo, Norway; 3 Kristianstad University Sweden, Sweden

**Keywords:** elderly people living at home, prepared meals, PCA

## Abstract

Prepared meals distributed by municipalities is a service to elderly people, or persons with health related impairments, who live in their own home, have difficulties preparing their own food and cannot meet their food requirements in any other way. This study aimed to provide a brief picture of how elderly people living at home perceive the food they receive through their municipal food service and what is important to them. The data was collected using questionnaires. 274 out of 276 participants answered the questionnaire (n=173 women 62% and n=101 man 37%). The data was analyzed using Principal Component Analysis (PCA).

The results showed that the elderly persons receiving meals through the service were often satisfied, especially with the size of the portions and the delivery time. Those who had been using the food delivery service for a longer time were not satisfied with the alternative dishes they were been offered. There was no significant difference between the views of either gender. Further, those who were receiving special food were, in general, unsatisfied with the meals delivered.

Development of the food distribution service by systematic quality insurance and interactive knowledge exchange between the producers and consumers seems to be a way to promote a more holistic and individual adjusted service. Evaluation of the municipal FD service is a powerful tool that can contribute to the development of this service.

The food service can be improved and consequently even the quality of life and health of its receivers. The present survey should be revisited and developed in order to detect differences between genders.

## 1. Background

Prepared meals distributed by municipalities in Sweden is a service to elderly people, or persons with health related impairments, who live in their own home, have difficulties preparing their own food and cannot meet their food requirements in any other way (Pajalic & Westergren, 2013). Swedish elderly care and the prepared meals service is publicly financed and a publicly provided service (Szebehely & Trydegård, 2012). The organization of food distribution (FD) in Sweden differs from that in other welfare states where the FD, is mostly organized by either private companies or voluntary organizations (Pajalic, Persson, Westergren, & Skovdahl, 2012). Further, each Swedish municipality has the responsibility to perform quality assurance for their social and care services. To help in this work they have various documents, tools and procedures adjusted to support the different activities (Joakim Ramsberg, Hanna Sjöberg, Therese Östh, & Sandsborg, 2006).

In Sweden, the municipality prepared meals are subsidized by taxes and it is not possible simply to request this service “carte blanche”. Requests for the service are subject to need assessment by public home care officers (Pajalic, 2013c). Of the approximately 291 municipalities in Sweden, 280 produce meals at municipally owned kitchens, while the remainder, who provide meals, outsource this service to private operators. Distributed meals comprised of mostly of lunches which corresponded to 30% (Akner, 2003) of the elderly people’s daily nutritional needs. Breakfast, snacks, bread, salad, beverages and evening meals need to be arranged for by the elderly themselves and is outside of the municipal service (Edfors, & Westergren, 2010; Pajalic, 2013a). The meals are delivered either in frozen form every week or freshly made daily. In most municipalities it is common to deliver freshly prepared warm food. In accordance with The Swedish National Food Agency (Bergström, 1998; Lantz & Svensson, 2002) the freshly made food should be delivered personally to the customer, that is to say “into their hands”, and not simply left at their door. Swedish municipalities have a statutory obligation to provide social and care service assistance to all persons in the municipality needing help. This statute is regulated by two acts: the Social Services Act (Grönwall & Holgersson, 2000) and the Health and Medical Services Act (Raadu, 2011). Each municipality may organize their FD services according to their own circumstances as neither of these two acts has detailed information regarding how the FD should be organized (Faxén Irving, Karlström, & Rothenberg, 2010). The consequence of the lack of detailed information is that there are 291 municipalities and 291 different ways of organizing the food service (FD) to the elderly service, resulting in unequal and non-comparable services (Pajalic & Westergren, 2013). A food service to the elderly and infirm is a minimum measure from society in order to prevent starvation among older and infirm persons who cannot cook their own food (Lagergren et al., 2004; Lammes, Torner, & Akner, 2009; Mattsson Sydner, 2002). No checks were made relating to the applicant’s nutrition status before the service was granted and there is no follow-up of how the FD service affects a person’s nutritional status over time (Pajalic, 2013b; Pajalic, Persson, Westergren, & Skovdahl, 2012). The question is whether the food service is optimal. The knowledge gained from this study can be a starting point for shaping municipal food services towards what the elderly consider most important and which can eventually affect their food intake and quality of life.

## 2. Objectives

This study aimed to provide a brief picture of how elderly people living at home perceive the food they receive through their municipal food service and what is important to them.

## 3. Ethical Considerations

The study has been approved by the regional ethical board (LU09/365). No personal information that would allow any data to be linked to individual participants was recorded.

## 4. Method

Data was collected, using questionnaires, during March 2009. The focus was on: gender (GEN), the length of time for receiving of FD (LEN), how many days the meals were distributed each week (DPW), how the participants experience the taste of the food (TAS), the variation of the dishes (VAR), if there were any alternative dishes on the menu (ALT), if any special diet was available (OTH), the satisfaction level with a special diet (SAT), the level of satisfaction with the delivery time of the meals (DEL), the size of the portions (SIZ), did relatives, service personnel or any others offer assistance during meal times (HEL), were evening meals or snacks available? There was also a possibility to offer suggestions regarding what in the FD service could be improved. The Principal Component Analysis (PCA) was used to analyse the correlation between the variables in the data (Jolliffe, 2005) and also the programming tool Umetrics SIMCA 13.0 (SIMCA, 2005; Umetrics, 2005). The components consisted of score vectors, t[i], describing all the variables used in the analysis and loadings vectors, p[i] that described how the variables are combined and which variables were important.

### 4.1 Context

The study was conducted in a medium-sized municipality situated in Sweden. Approximately 276 elderly people used the municipal food distribution during the autumn of 2009. The number of elderly persons receiving food by FD in Sweden (about 9, 8 million inhabitants) is estimated to 60 000. However this number varies as some of the elderly people chose to receive the FD service for only certain days of the week and arranged their lunch in another way. The meals that were distributed were lunches while breakfast and eventual evening meals needed to be arranged by the client. The lunches were freshly produced at a municipal kitchen and delivered personally to the elderly persons as a lunch box. Twice a year the municipality provided the possibility of an evaluation by offering the clients a questionnaire to complete and return. The focus was on how the elderly perceive the food they receive with the aim of adjusting the service to better meet the clients requirements.

### 4.2 Participants and Analyses

Totally, 276 questionnaires were sent to municipal FD receivers. The 62 % (n=173 women) and 37% (n=101 man) answered the questionnaire and 1% (n=2 elderly persons) did not reply (Table 1).

**Table 1:** Overview of participants’ responses to the questionnaire

Variables	yes	often	medium	seldom	no
GEN	101				173
LEN	132		86		38
DPW	218		28		26
TAS	84	158		23	0
VAR	96	146		21	2
ALT	148		94		16
OTH	38				208
SAT	8	24		3	0
DEL	230		20		17
SIZ	228		17		18
HEL	123				153

The variables from the surveys were given in descriptive form as: yes, no, seldom or often. The variable was quantified into values between 0 and 1 in order to compare different observations using the PCA model.

The weighting was as follows: yes was 1, no 0, often 0,66 and seldom 0,33. In questions on length of time of receiving (LEN) and how many times per week (TPW) three alternatives were given that were scaled as 1, 0,5 and 0.

Judgment of variable importance was not considered during the weighting of variables and linear weight applied, as is shown in Table 2.

**Table 2:** Weighting of variables

		Weighting
GEN	Male	1
	Female	0
LEN	>2 years	1
	0,5-2 years	0,5
	<0,5 y	0
DPW	3	0
	4-5	0,5
	6-7	1
TAS	always	1
	often	0,67
	seldom	0,33
	never	0
VAR	always	1
	often	0,67
	seldom	0,33
	never	0
ALT	yes	1
	dont know	0,5
	no	0
OTH	yes	1
	no	0
SAT	always	1
	often	0,67
	seldom	0,33
	never	0
DEL	yes	1
	to late	0,5
	too early	0
SIZ	suitable	1
	too large	0,5
	too small	0
HEL	yes	1
	no	0

The description of the model was made in the following: number of variables: 5; number of observations: 11; missing values: 22 (40%). All variables were scaled to Unit Variance. The model is described with two significant components (Table 3). The acumulated goodnes of fit in X, R2X(cum) is 0.993.

**Table 3:** Model summary

Component	R2X	R2X(cum)	Eigen value	Q2	Limit	Q2(cum)	Significance
1	0,658	0,658	7,24	0,376	0,161	0,376	R1
2	0,229	0,888	2,52	-0,307	0,175	0,313	NS
3	0,105	0,993	1,16	0,599	0,192	0,724	R1

## 5. Results

The R2X shows the cumulative percent of the variation of the X variable explained by the model and is the measure of fit, i.e. how well the model fits the data. The Q2 (cum) shows the cumulative percent of the variation of the X variable predicted by the model according to cross validation. Q2 tells how well the model predicts the variable.

**Figure 1:**
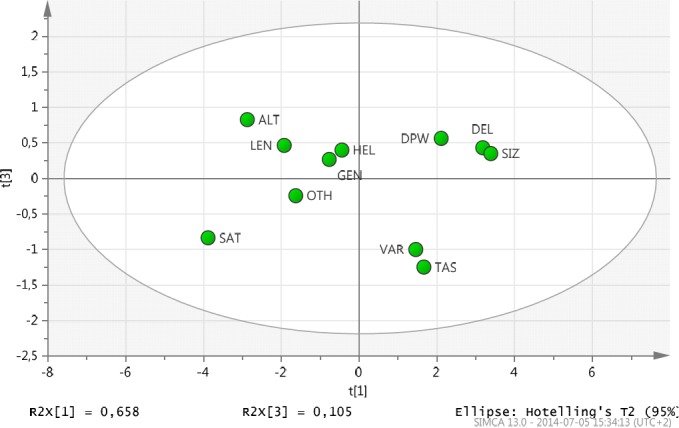
Model Results, Scores t[1] vs. t[3]

The score t[1] (first component) explains the largest variation of the X space, followed by t[3] (Figure 1). Hence the scatter plot of t[1] vs. t[3] is a window in the X space displaying how the observations are situated with respect to each other. Observations close to each other are similar observations far away from each other are dissimilar. Hotelling´s T2 is a measure of how far away an observation is from the center of the PCA model.

**Figure 2:**
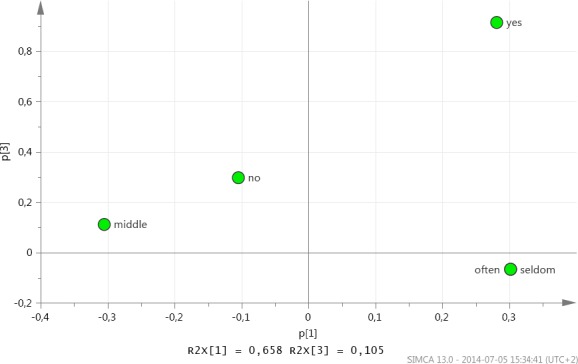
Model results, Loadings p[1] vs. p[3]

The scores are weighted averages of the variables with weightings p[1] in the first dimension and p[3] in the second dimension (Figure 2). Hence p[1] vs. p[3] displays how the X variables correlate with each other and contribute to the model. Points that are far away from the origin have a strong impact on the model, whereas points that are closer to the centre have a weaker influence.

By analysing the score plot and its combination with the loading plot, the following conclusions are possible:

- Those who received meals often (DPW) are satisfied with the portion size and delivery time but those who have received municipal FD over a longer time were not satisfied with the alternatives (ALT) they have been offered. That could be explained by the municipal FD receivers being tired of the same menu over time.
- Those who receive special food (OTH) are not satisfied with the taste (SAT)
- Gender (GEN) and assistance (HEL) have shown no significance in the model

## 6. Discussion

### 6.1 Discussion of Method

In order to get better outcomes from the study, the questionnaire must be improved, particularly by avoiding questions which can give responses that could covariate. The analysis would give better correlations if the answers were valued by numbers one to ten instead of descriptions as often or seldom, or yes and no.

### 6.2 Discussion of Results

The present study showed that there is no significance between the genders. This results were in contradiction to another study that showed that restrictions on living an independent life was an indicator of social isolation that could be associated to food choice and that there were women with lower socio economic status who were at risk of developing complications related to food intake (Locher et al., 2005). In contrast to this it was shown that women gave higher marks for health pleasure and convenience in their evaluations of healthy meals (Rappoport, Peters, Downey, McCann, & Huff-Corzine, 1993). Gender differences were more pronounced between socio-economic groups. There were no gender differences concerning the intake of fish in terms of health. Women can be viewed as innovators, as well as mediators, for change towards a healthier diet (Fagerli & Wandel, 1999). Several significant differences between men and women were identified: views on food and health; the ethical dimensions of food production and food selection; nutritional attitudes and choices; dietary change and body image. Two distinctive patterns that emerged were: virtuous and robust types among the women (Beardsworth et al., 2002). Women across the world are more convinced than men that dietary choices are important (Wardle et al., 2004). Low education levels and socio-economic status among the frail elderly, no matter their gender, were identified as factors that should influence the future quality of municipal food distribution and food quality (Szebehely & Trydegård, 2012). The reason for why the evaluation of the results in the present study showed no significance between them could perhaps be in the formulation of the questionnaire.

The results in the present study showed that elderly persons who are receiving meals through the FD service are satisfied, especially with the portion size and delivery time. At the same time those who have been receiving FD over a longer time are not satisfied with the alternatives (ALT) they have been offered. The Kim et al (2010) study showed that satisfaction with a foodservice is directly correlated to positive social interaction between the elderly and those who distribute the food (Kim, Joung, Yuan, & Huffman, 2010). Further, those who deliver the food are important for the creation of a positive social contact and in that way help minimise feelings of loneliness and isolation among their clients (Pajalic, Persson, Westergren, Berggren, & Skovdahl, 2012; Winterton, Warburton, & Oppenheimer, 2013). Similar results were shown in the Tomstad et al (2013) study that showed that dialogue between health care professionals and elderly food recipients can stimulate the consciousness among the elderly regarding the importance of nutrition for health and the prevention of under-nourishment (Tomstad, Söderhamn, Espnes, & Söderhamn, 2013). The food preferences and general satisfaction of the municipal food distribution recipients was influenced by good food quality and the positive response by the FD staff (Lirette, Podovennikoff, Wismer, & Tondu, 2007; Roberts, Wolfson, & Payette, 2007). Prevention of under-nourishment includes individually tailored diet information from dieticians, and it is important that this information is based on each individual’s health status. This tailored information is essential for successful nutrition management for the elderly (Hirakawa, Kimata, & Uemura, 2013). Gough and Conner (2006) found that men were more skeptical towards recommendations about eating health food due to the fact that health food often does not taste and does not satisfy their hunger.

Their resistance can be explained as a negative reaction to recommendations from others because they experience their situation as a reduction of their choice and freedom. Contrary to this, they showed that the elderly take diet recommendations into consideration due to the diet’s consequence on their health and mortality (Gough & Conner, 2006). Another study (Lee & Joo, 2012), showed that information in combination with the experience of security had a positive correlation with the receivers satisfaction of the food service (Lee & Joo, 2012). The basis for complaints among the elderly was often the aggravation for them based on their dependent situation (Gough & Conner, 2006). The food service needs to be held as important in relation to the life and experience of the elderly and the food service needs to be better adjusted and more focused on people’s lifestyles and behaviour (Sydner & Fjellstrom, 2005). Further, a monotonous variety in food dishes leads to decreased food intake. Repeated consumption of the same food during a week resulted in decreased satisfaction and increased monotony over time. This was specifically experienced by persons who received the same food daily without any possibility to choose between various tastes (Meiselman, de Graaf, & Lesher, 2000). Frequent and repeated exposure to the same diet can lead to decreased stimulus and despondency. When the same food was eaten often it was shown to influence the experience of satisfaction and that the daily consumption of otherwise favorite dishes can become monotonous (Hetherington, Pirie, & Nabb, 2002; Hughes, Bennett, & Hetherington, 2004). Dietary monotony and food desires in the elderly can result in a non-adequate diet. If the elderly are not stimulated to eat a wider variation of food it can also lead to their receiving inadequate nutrition (Pelchat & Schaefer, 2000).

The results in the present study showed that those who receive special food (OTH) are not satisfied with its taste (SAT). This can be explained by the findings in a study by Winter Falk and Sobal (1996) who focused on food choices for the elderly whose tastes were strongly influenced by their pattern of food behavior which had been formed during their childhood. Further, social context, sensory perception, monetary considerations, convenience and physical well-being were important values upon which the elderly made their food choices. The elderly dealt with food choice with strategies such as routines, substitution, limitations and exclusion (Winter Falk, Bisogni, & Sobal, 1996). Dean et al. (2009) showed that income, health status and living arrangements affected a person’s level of dietary variety. The variety of food choices among the elderly depended on material resources, their appetite, their food knowledge, the distance to the shops, their access to high-quality products, kitchen facilities, and access to good food service producers and practical support from others. All these factors contributed to how varied a diet the elderly had (Dean, Raats, Grunert, & Lumbers, 2009). Any food service or help with food that influences the diet of the elderly and their food options should be innovative and individually adjusted and aimed to meet the complex nutritional needs of the ageing population (Winterton et al., 2013). Further, the best practices for the food delivery services and those involved in this service, such as the home care professionals, should be based on the continuous development of nutritional knowledge by the professionals and include the experience of consumers i.e. the elderly (Buchanan et al., 2009; Pajalic, Persson, Westergren, & Skovdahl, 2012). This was confirmed in a study by Wilson et al. (2004) that showed that social and organisational factors, and the usage of food shops have a direct effect on dietary variety among the elderly population (Wilson, Alexander, & Lumbers, 2004). Lilley (1996) showed that the elderly who have good nutritional knowledge eat well, but that person’s ≥ 75 years often lacked variety in their diet. Elderly people in urban areas often use public transport to shop for food and therefore have choices of food and diet variety, while those in rural areas often have poor access to public transportation which limits their food choices Further constraints, resulting in limited food choice for the elderly, are their health status, possible ill-health, mobility and transport, and also other practical issues such as being near to shops and further their demographic status such as their social class and other factors that can cause isolation (Lilley, 1996). Herne (1995) showed that the combination of physical and mental health and social factors of class and income and nutritional education, influence people’s satisfaction with their meals. For example the elderly did not feel that nutrition education was relevant for them and that their problems related to food intake were merely a natural consequence of getting older. Nutrition education needs to pay attention to the needs of the elderly because offering nutrition information is essential to promote the means for them to change and adjust their diet according to their needs (Herne, 1995). McKie (1999) described how the elderly developed strategies and tried to adjust their food needs by using local restaurant options where available. Further they may have had to adapt their food preferences due to their mobility and possible access difficulties when visiting food shops (McKie, 1999). Wylie et al. (1999) showed that the elderly with restricted mobility may be unable to consume an optimal nutritional intake due to their health and social factors which can affect their choice of food, satisfaction with food and their nutritional intake (Wylie, Copeman, & Kirk, 1999).

Based on the study results above, other questions arise such as: is the representation by participants from an urban area in the present study any higher than those from a rural area? Do they have a better possibility to pay for additional service then the one from the municipality? Do they have more help from others, and do they eat at restaurants? These questions are important to focus on in the any future study. In conclusion, the knowledge regarding elderly people’s needs for an individually adjusted food service has increased dramatically during the last decade. Despite this, undernourishment is still common. Insufficient food intake in relation to requirement is one of most important reasons for undernourishment. Therefore, care and service for elderly people must be based on an assessment of their risk for undernourishment and include detailed follow up of all the actions taken in this question. For those societies whose demographics change fast, it is important to take into consideration that there is a great need to meet the elderly population’s nutrition needs.

### 6.3 Key Findings

- Development of the food distribution service by systematic quality insurance and interactive knowledge exchange between the producers and consumers seems to be a way to promote a more holistic and individual adjusted service
- Evaluation of the municipal FD service is a powerful tool that can contribute to the development of this service. The food service can be improved and consequently even the quality of life and health of its receivers.
- The present survey should be revisited and developed in order to detect differences between genders

**Competing Interest**

The authors declare that they have no competing interests and non-financial competing interests

**Author’s Contribution**

Study design and data ZP; statistical analysis and interpretation of data OP; drafting and critical review of the manuscript ZP & OP; who have also given their approval of the version to be published.
